# Two Plant Viral Suppressors of Silencing Require the Ethylene-Inducible Host Transcription Factor RAV2 to Block RNA Silencing

**DOI:** 10.1371/journal.ppat.1000729

**Published:** 2010-01-15

**Authors:** Matthew W. Endres, Brian D. Gregory, Zhihuan Gao, Amy Wahba Foreman, Sizolwenkosi Mlotshwa, Xin Ge, Gail J. Pruss, Joseph R. Ecker, Lewis H. Bowman, Vicki Vance

**Affiliations:** 1 Department of Biological Sciences, University of South Carolina, Columbia, South Carolina, United States of America; 2 Plant Biology Laboratory, The Salk Institute for Biological Studies, La Jolla, California, United States of America; 3 Genomic Analysis Laboratory, The Salk Institute for Biological Studies, La Jolla, California, United States of America; University of California Riverside, United States of America

## Abstract

RNA silencing is a highly conserved pathway in the network of interconnected defense responses that are activated during viral infection. As a counter-defense, many plant viruses encode proteins that block silencing, often also interfering with endogenous small RNA pathways. However, the mechanism of action of viral suppressors is not well understood and the role of host factors in the process is just beginning to emerge. Here we report that the ethylene-inducible transcription factor RAV2 is required for suppression of RNA silencing by two unrelated plant viral proteins, potyvirus HC-Pro and carmovirus P38. Using a hairpin transgene silencing system, we find that both viral suppressors require RAV2 to block the activity of primary siRNAs, whereas suppression of transitive silencing is RAV2-independent. RAV2 is also required for many HC-Pro-mediated morphological anomalies in transgenic plants, but not for the associated defects in the microRNA pathway. Whole genome tiling microarray experiments demonstrate that expression of genes known to be required for silencing is unchanged in HC-Pro plants, whereas a striking number of genes involved in other biotic and abiotic stress responses are induced, many in a RAV2-dependent manner. Among the genes that require RAV2 for induction by HC-Pro are *FRY1* and *CML38*, genes implicated as endogenous suppressors of silencing. These findings raise the intriguing possibility that HC-Pro-suppression of silencing is not caused by decreased expression of genes that are required for silencing, but instead, by induction of stress and defense responses, some components of which interfere with antiviral silencing. Furthermore, the observation that two unrelated viral suppressors require the activity of the same factor to block silencing suggests that RAV2 represents a control point that can be readily subverted by viruses to block antiviral silencing.

## Author Summary

RNA silencing is an important antiviral defense in plants, and many plant viruses encode proteins that block RNA silencing. However, the mechanism of action of the viral suppressors is complex, and little is known about the role of host plant proteins in the process. Here we report the first example of a host protein that plays a required role in viral suppression of silencing—a transcription factor called RAV2 that is required for suppression of silencing by two different and unrelated viral proteins. Analysis of plant gene expression patterns shows that RAV2 is required for induction of many genes involved in other stress and defense pathways, including genes implicated as plant suppressors of silencing. Overall, the results suggest that RAV2 is an important factor in viral suppression of silencing and that the role of RAV2 is to divert host defenses toward responses that interfere with antiviral silencing.

## Introduction

Plants have a complex interconnected system of defense and stress pathways [1],[2] that receives incoming stimuli, transduces the signal and initiates the appropriate response. The process is orchestrated by a variety of plant hormones and small signaling molecules, and the final shape of the response is refined by crosstalk among different pathways in the network. Evidence emerging over the last decade has made it clear that RNA silencing and endogenous small RNA pathways constitute a major response to a variety of biotic and abiotic stresses [3],[4],[5]. Surprisingly, however, although many of the components of the silencing machinery are known, little is yet known about how silencing is regulated or how it is integrated into the network of other defense and stress pathways.

RNA silencing is a sequence specific RNA degradation mechanism that serves an important antiviral role in plants [6]. Antiviral silencing is triggered by double stranded RNA (dsRNA) that arises during virus infection. The dsRNA trigger is processed by DICER-LIKE (DCL) ribonucleases into primary short interfering RNAs (siRNAs), which incorporate into an ARGONAUTE (AGO) protein-containing effector complex and guide it to complementary target RNAs. The destruction of target RNAs can be amplified via a process called transitive silencing, in which the target RNA serves as template for host RNA-dependent RNA polymerases (RDRs) to produce additional dsRNA that is subsequently processed into secondary siRNAs. In addition to these RDRs, a number of other genes, including *DCL2*, *AGO1* and *SUPPRESSOR OF GENE SILENCING 3* (*SGS3*), are required for transitive silencing, but not for primary silencing [7],[8],[9]. The primary and transitive silencing pathways work together to limit the accumulation of viral RNAs during both the initial and systemic phases of infection.

In addition to antiviral silencing and related pathways that target invading nucleic acids, there are endogenous small RNA pathways that regulate gene expression by directing cleavage of target RNA, inhibition of mRNA translation, or modification of chromatin structure. The best studied of the endogenous small RNAs are the microRNAs (miRNAs), which play major roles in development and in response to a variety of stresses [10],[11],[12]. Although different small RNA mediated pathways have unique genetic requirements, all make use of an overlapping set of genes for their biogenesis (four *DCL* genes) and function (ten *AGO* genes), and there is growing evidence that these pathways are interconnected and compete with one another. For example, *DCL1*, the Dicer that produces most miRNAs, represses antiviral silencing by down-regulation of *DCL3* and *DCL4* [13] and, when over-expressed, blocks silencing induced by a sense transgene [14]. In addition, many viral suppressors of RNA silencing also interfere with the biogenesis and/or function of endogenous small RNAs such as miRNAs and trans-acting small interfering RNAs (tasiRNAs) [15],[16]. However, the mechanisms that regulate and integrate the various small RNA pathways are just beginning to be elucidated.

Plant viruses have evolved a variety of effective counter-defensive strategies to suppress silencing. Numerous plant viruses encode proteins that block some aspect of RNA silencing [15],[16]. These viral proteins are highly diverse in primary sequence and protein structure, though they may share certain mechanistic features. For example, the ability to bind small RNAs is a feature of many viral suppressors of silencing, including the two used in the present work. Indeed, it has been proposed that most viral suppressors of silencing work by binding and sequestering small RNAs, thereby blocking their activity [17],[18]. However, the physiological significance of small RNA binding is not yet clear in many cases [6], and some suppressors manipulate silencing via interaction with host proteins that are either components of the silencing machinery [19],[20],[21],[22] or proposed regulators of the pathway [23]. Thus, the mechanism of action of viral suppressors is likely both diverse and complex and is not yet fully understood.

Our studies have focused on understanding the mechanism of action of HC-Pro, a potent viral suppressor of silencing that blocks both primary and transitive silencing. Our approach has been to identify host proteins that physically interact with HC-Pro and examine the effect of altering the levels of these proteins on both RNA silencing and the ability of HC-Pro to block silencing [23]. Using this approach, we find that RAV2/EDF2 (hereafter referred to as RAV2), an HC-Pro-interacting protein that is a member of the RAV/EDF family of transcription factors, is required for suppression of silencing not only by potyvirus HC-Pro, but also by carmovirus P38, the silencing suppressor from a virus family unrelated to potyviruses. Interestingly, RAV2 is required exclusively for blocking the activity of primary siRNAs, whereas suppression of transitive silencing and effects on the endogenous microRNA pathway are RAV2-independent. Whole genome tiling microarray experiments were used to characterize HC-Pro-mediated changes in host expression and identify which, if any, were RAV2-dependent. The results raise the interesting possibility that HC-Pro-suppression of silencing is not caused by decreased expression of genes that are required for silencing, but instead, by induction of stress and defense pathways that interfere with antiviral silencing.

## Results

### Ectopic expression of a RAV/EDF transcription factor delays the onset of transgene-induced RNA silencing in tobacco

In previous work we used a yeast two-hybrid screen to identify *Nicotiana tabacum* proteins that interact with Tobacco Etch Potyvirus (TEV) HC-Pro [23]. One of the proteins identified in this way was named ntRAV because of its relatedness to the *Arabidopsis thaliana* RAV/EDF family of transcription factors. The RAV/EDF protein family has six members, and these are unique among transcription factors in having two unrelated DNA binding domains (AP2 and B3) [24]. Members of this family are responsive to numerous biotic and abiotic stresses [25],[26],[27],[28] and are inducible by the plant hormone ethylene [29], which controls many aspects of plant physiology, including defense against pathogens [30],[31].

**Figure 1:**
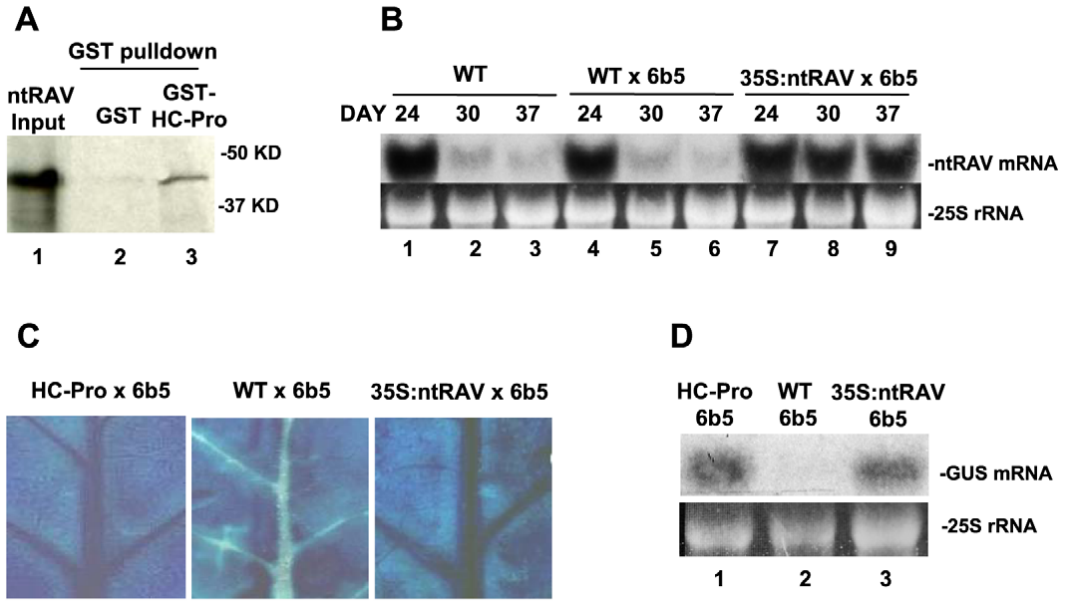
ntRAV Interacts with HC-Pro and Delays the Onset of Sense Transgene Silencing when Over-expressed in Tobacco. (**A**) Tobacco ntRAV interacts with TEV HC-Pro in *in vitro* pulldown experiments. 35S-labelled ntRAV co-purifies with HC-Pro-GST (lane 3), but not with GST (lane 2). Lane 1 shows the amount of input 35S-labelled ntRAV protein used in the pulldown experiments. (**B**) The accumulation of *ntRAV* mRNA at 24, 30 and 37 days after germination in whole leaves of wild type (WT) tobacco plants (lanes 1–3), plants heterozygous for the silenced *6b5* GUS transgene (WT X 6b5) (lanes 4–6), and plants heterozygous for the silenced *6b5* GUS transgene and expressing the *35S:ntRAV* transgene (lanes 7–9). (**C**) Histochemical staining of leaves from HC-Pro X 6b5 (left panel), WT X 6b5 (center panel) and 35S:ntRAV X 6b5 leaves (right panel) at 26 days after germination. (**D**) GUS mRNA levels in the veins of leaves of HC-Pro X 6b5 (lane 1), WT X 6b5 (lane 2) and 35SntRAV X 6b5 plants (lane 3) at 26 days after germination.

*In vitro* pull-down experiments were used to confirm a physical interaction between TEV HC-Pro and ntRAV. ^35^S-methionine-labeled ntRAV produced in a coupled *in vitro* transcription/translation system co-purified with an HC-Pro-GST fusion protein isolated from recombinant bacteria, but not with GST alone (Fig. 1A and see also Fig. S1 and Text S1). This result validates the HC-Pro-ntRAV interaction initially identified in the yeast two-hybrid system.

To determine if *ntRAV* plays a role in RNA silencing, we evaluated the effect of ntRAV over-expression on transgene-induced silencing. In tobacco, *ntRAV* is normally expressed at high levels throughout fully expanded healthy leaves of young plants, but expression decreases greatly starting at about 24 days after germination (Fig. 1B, lanes 1–6). In contrast, a tobacco line that ectopically expresses *ntRAV* from the constitutive Cauliflower mosaic virus (CaMV) 35S promoter maintains high level expression of *ntRAV* (Fig. 1B, lanes 7–9). We crossed the *35S:ntRAV* transgenic line, as well as wild type and HC-Pro-expressing control lines, to the well-characterized tobacco transgenic line 6b5 [32], which is post-transcriptionally silenced for a transgene encoding β-glucuronidase (GUS). Silencing of the *GUS* locus in line 6b5 reinitiates every generation, starting in the vascular tissue of the oldest leaves and then spreading throughout the leaf. The expression of *GUS* in F_1_ progeny of these crosses was assayed histochemically in leaves (Fig. 1C) and by northern blots of RNA from the vascular tissue (Fig. 1D) at 26 days after germination. In these young plants, ectopic expression of *ntRAV* blocked silencing of *GUS* in vascular tissue of fully expanded, healthy leaves about as well as HC-Pro (Fig. 1C and D). However, unlike HC-Pro, which completely blocks silencing over the lifetime of the plant, ectopic expression of *ntRAV* only delayed the onset of silencing, and *GUS* was eventually silenced throughout the leaf (data not shown). These results, together with those showing a physical interaction between ntRAV and TEV HC-Pro proteins, raised the possibility that ntRAV plays a role in HC-Pro-mediated suppression of silencing.

### Experiments in the model plant, *Arabidopsis thaliana*

To further investigate the role of *ntRAV* in HC-Pro suppression of silencing, we switched from tobacco to *Arabidopsis thaliana*, in order to take advantage of the numerous genetic tools available in that model system. Our experiments focused on a *RAV* gene family member closely related to the tobacco *ntRAV*, Arabidopsis *RAV2* (At1g68840), which had already been cloned and characterized, and for which a validated T-DNA insertional knockout line was available [29]. The change in experimental system also necessitated a change from the HC-Pro encoded by TEV to that encoded by turnip mosaic virus (TuMV), a related potyvirus that infects *Arabidopsis*. Like the TEV HC-Pro transgene in tobacco, expression of the TuMV HC-Pro in transgenic *Arabidopsis* plants has been shown to suppress both virus- and transgene-induced RNA silencing [14],[33]. The TuMV HC-Pro transgenic line used in our experiments expresses HC-Pro at a high level and is highly phenotypic [14].

**Figure 2:**
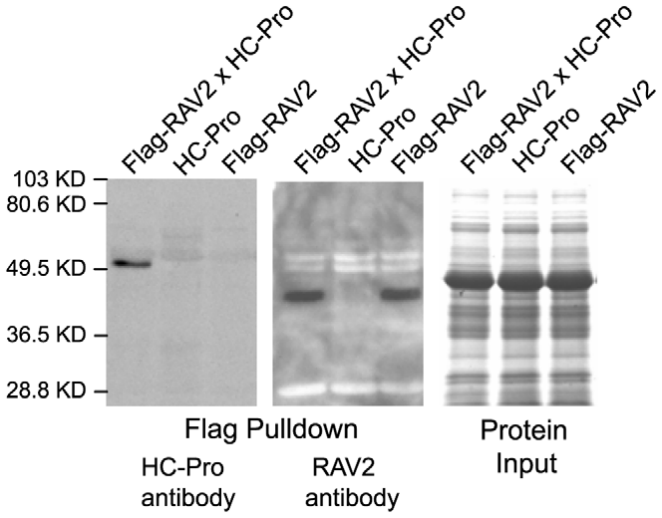
*In vivo* Interaction of RAV2 and TuMV HC-Pro in Arabidopsis. Proteins isolated from plants expressing either FLAG-tagged RAV2 (Flag-RAV2) alone, TuMV HC-Pro alone or both Flag-RAV2 and TuMV HC-Pro were incubated with anti-FLAG agarose beads. The bound protein was fractionated on acrylamide gels and subjected to western blot analysis using either HC-Pro antiserum (left panel) or RAV2 antiserum (center panel). The far right panel shows the relative input amounts of protein used in the pulldown experiments as determined by Coomassie blue staining.

We used *in vivo* pull-down experiments to determine whether the TuMV HC-Pro and RAV2 proteins interact, as would be expected if *RAV2* were a functional homolog of *ntRAV*. In these experiments, the homozygous *rav2* knockout line [29] was transformed with a construct designed to express a transgene encoding FLAG-tagged RAV2. A transformant that expressed the *FLAG-RAV2* transgene was crossed to our TuMV HC-Pro transgenic line [14], and expression of both transgenes in the F_1_ offspring was confirmed by RNA gel blot analysis (data not shown). Pull-down experiments using antiserum specific to the FLAG tag, followed by western blot analysis, showed that TuMV HC-Pro co-immunoprecipitates with the Flag-tagged RAV2 (Fig. 2), indicating that RAV2 and TuMV HC-Pro interact *in planta* in *Arabidopsis*. This result confirms that *RAV2* is a functional homolog of *ntRAV* and also provides evidence that the interaction between potyviral HC-Pro and host RAV-like transcription factors is a conserved feature of these proteins.

### *RAV2* is required for HC-Pro suppression of virus induced gene silencing (VIGS)

**Figure 3:**
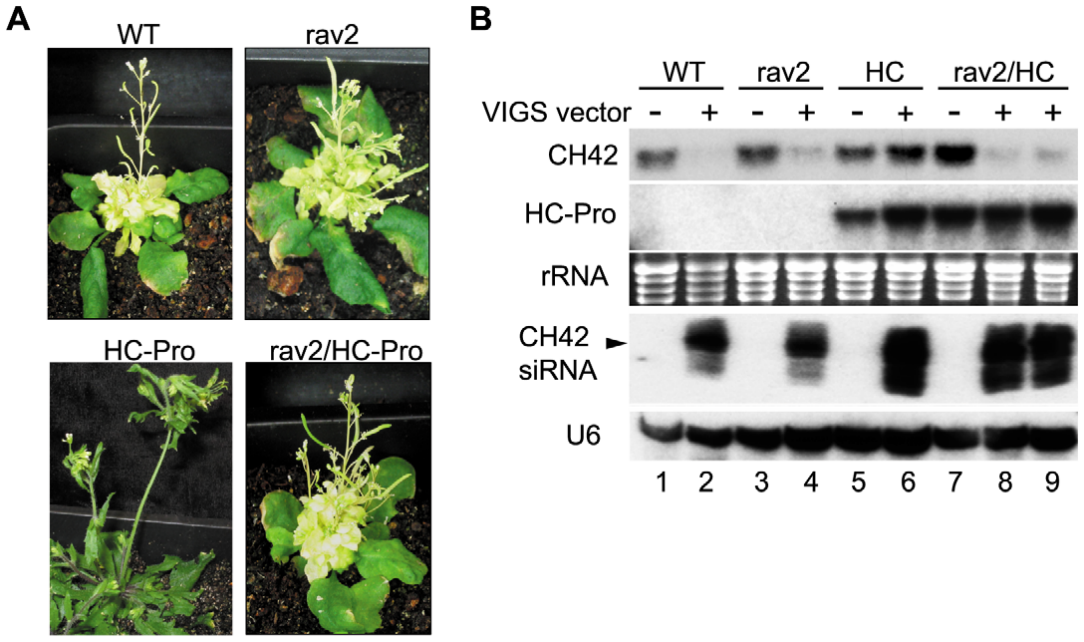
*RAV2* is Required for HC-Pro Suppression of Virus Induced Gene Silencing (VIGS). (**A**) Phenotype of plants bombarded with CaLCV vector carrying a portion of the endogenous *CH42* gene. VIGS of CH42 results in pronounced yellowing in wild type or *rav2* knockout plants (upper left and right panels, respectively). Plants expressing HC-Pro are suppressed for VIGS and therefore remain green (lower left panel); whereas HC-Pro plants in the *rav2* knockout background fail to block silencing and display yellowing typical of wild type plants (lower right panel). (**B**) RNA gel blot analysis of *CH42* mRNA, HC-Pro and *CH42* siRNA levels in wild type (lanes 1 and 2), rav2 knockout (lanes 3 and 4), HC-Pro plants (lanes 5 and 6) and HC-Pro plants in the rav2 background (lanes 7–9) either uninfected (lanes 1, 3, 5 and 7) or after bombardment with the CH42 VIGS vector (lanes 2, 4, 6, 8 and 9). Ethidium staining of rRNA is shown as the loading control for the high molecular weight blots and the hybridization signal for U6 is shown as the loading control for the small RNA blot. The migration of 24 nt siRNAs is marked by an arrow.

Our initial experiments to examine the role of *RAV2* in HC-Pro suppression of silencing focused on VIGS. These experiments used the well characterized geminivirus silencing vector, cabbage leaf curl virus (CaLCV), which carried a portion of the endogenous *CHLORATA42* (*CH42*) gene [34]. CH42 is required for chlorophyll accumulation, and VIGS of *CH42* in wild type plants results in extensive chlorosis and marked reduction in the level of *CH42* mRNA. These changes are accompanied by a pronounced accumulation of 24-nt siRNAs that derive from the *CH42* sequences within the viral vector [14],[34]. HC-Pro transgenic plants become infected when bombarded with the *CH42* VIGS vector and, although high levels of siRNAs accumulate in the plants, the *CH42* gene is not silenced as evidenced by accumulation of *CH42* mRNA and the absence of chlorosis [14]. To determine if *RAV2* is required for HC-Pro suppression of VIGS, plants expressing HC-Pro in either the wild type or the *rav2* knockout background, along with control plants, were bombarded with the *CH42* VIGS vector. Wild type control plants as well as *rav2* knockout plants exhibited chlorosis of infected tissues (Fig. 3A, top two panels) accompanied by reduction in *CH42* mRNA levels and the concomitant accumulation of siRNAs, as expected for VIGS (Fig. 3B, lanes 1–4). HC-Pro transgenic plants were suppressed for VIGS of *CH42*, remaining green (Fig. 3A, bottom left panel) and accumulating wild type levels of *CH42* mRNA as previously reported (Fig. 3B, lanes 5 and 6). In contrast, HC-Pro transgenic plants in the *rav2* knockout background were competent for VIGS of CH42 as evidenced by systemic chlorosis (Fig. 3A, bottom right panel) accompanied by reduction in *CH42* mRNA levels (Fig. 3B, lanes 7–9). This result indicates that *RAV2* is required for HC-Pro suppression of VIGS.

### *RAV2* is required for HC-Pro-suppression of the primary, but not the transitive, branch of hairpin transgene-induced RNA silencing

**Figure 4:**
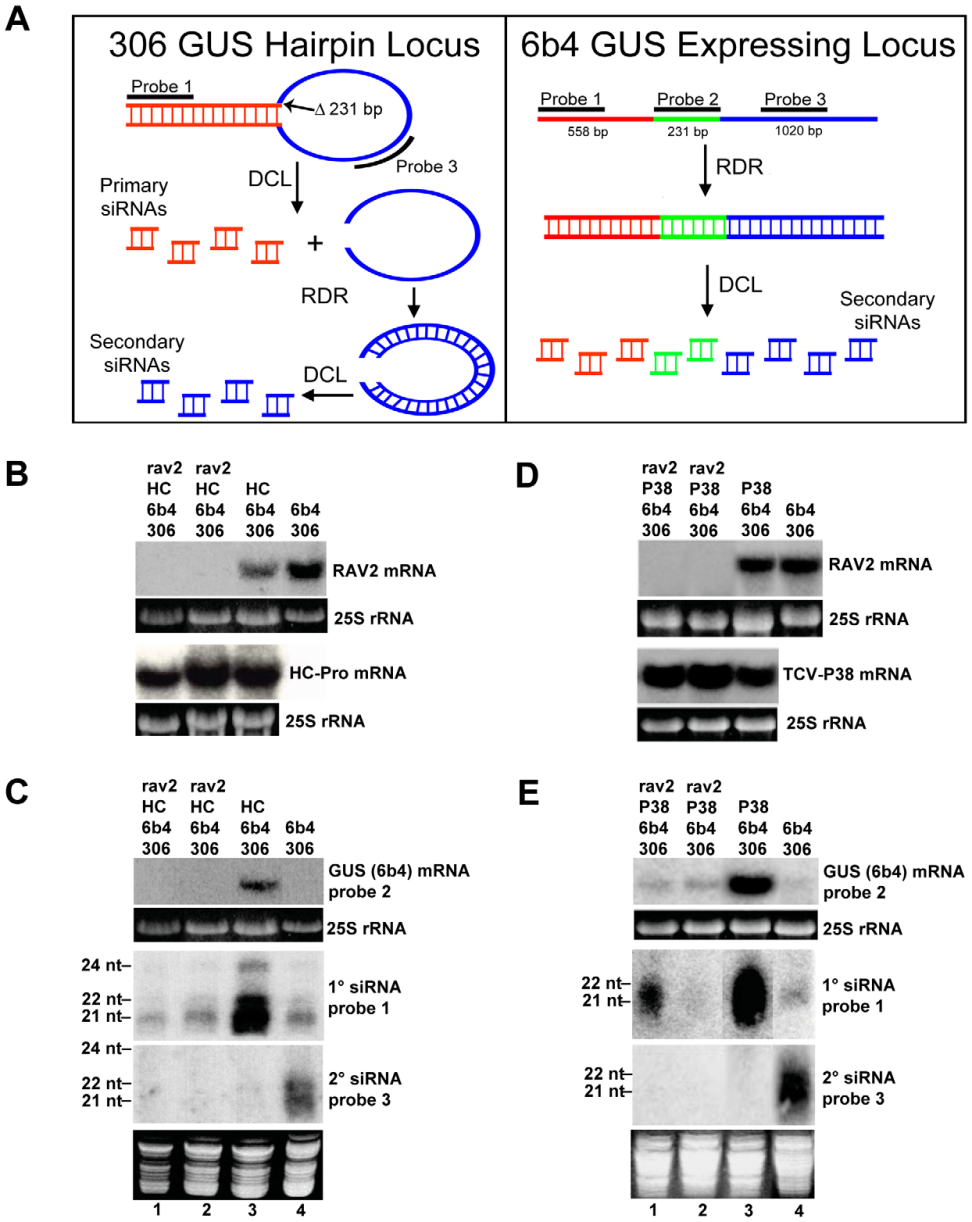
*RAV2* is Required for Suppression of Hairpin Transgene Silencing by Two Unrelated Viral Suppressors. (**A**) Diagrams showing the structures of the *6b4* and *306* transgene loci. The *6b4* locus is an expressing locus which encodes a functional GUS protein. The *306* locus produces a GUS hairpin RNA that acts *in trans* to silence the *6b4* locus. The locations of the hybridization probes used in parts **B**, **C** and **D** are indicated. (**B and D**) The accumulation of RAV2, TCV-P38 and/or TuMV HC-Pro mRNA in plants of the genotypes indicated at the top of the lanes. (**C and E**) The top panel of each shows the accumulation of *6b4* GUS mRNA in plants of the genotypes indicated at the top of the lanes, and the bottom two panels show the accumulation of primary and secondary siRNAs in the same samples. The size of 21-, 22- and 24-nt marker RNAs are indicated to the left of the small RNA panels and the probes used are indicated to the right of each panel.

To examine the role of *RAV2* in HC-Pro-suppression of transgene silencing, we used a well-characterized system in which silencing occurs through both the primary and transitive branches of the silencing pathway [7],[35]. This system is composed of two transgenes, the *306* and *6b4* loci (Fig. 4A). The *6b4* locus encodes an expressing GUS transgene that includes the entire GUS coding sequence, while the *306* locus encodes a hairpin construct designed to silence GUS expression. The GUS sequence in the *306* locus has a 231 nucleotide deletion in the coding region (Fig. 4A, shown in green) so that RNAs originating from the *6b4* transcript can be unambiguously distinguished. The primary and transitive branches of silencing can be easily differentiated in this system. Basically, primary siRNAs derive only from the stem of the *306* hairpin transcript (Fig. 4A, shown in red, probe 1), whereas secondary siRNAs arise from either locus during an RDR6-dependent process called transitive silencing. In the case of the *306* transgene, siRNAs that arise from the loop of hairpin transcript are secondary siRNAs (Fig. 4A, shown in blue, probe 3). In contrast to the *306* hairpin transcript, the *6b4* mRNA produces only RDR6-dependent secondary siRNAs (Fig. 4A, shown in red, green and blue; [7]. Thus, in the *306*/*6b4* system, *6b4* mRNA can be degraded by two mechanisms. It can be targeted by a RISC complex directed by siRNAs, or it can be a substrate for RDR6, producing dsRNA that is subsequently processed by DCL to produce secondary siRNAs via transitive silencing. HC-Pro suppresses silencing in the *306*/*6b4* system, but has different effects on primary and secondary siRNAs: accumulation of secondary siRNAs is eliminated, as shown by the failure to detect any siRNAs when using either probe 2 or probe 3 [7]. In contrast, high levels of primary siRNAs accumulate, but are unable to mediate degradation of the *6b4* target RNA [7].

To determine if *RAV2* is required for HC-Pro suppression of hairpin transgene silencing, we crossed the homozygous *rav2* knockout line to a transgenic line homozygous for the *306* and *6b4* loci and hemizygous for the TuMV *HC-Pro* locus. F_1_ offspring of this cross were allowed to self-fertilize, producing an F_2_ population that was segregating for all four loci. F_2_ plants were genotyped, and individuals containing the *306/6b4/HC-Pro* loci in the homozygous *rav2* mutant background were identified, along with control plants containing all three loci in the wild type *RAV2* background. The absence of *RAV2* mRNA in *rav2* knockout plants was verified by RNA gel blot analysis (Fig. 4B). Initial analysis of the *306/6b4/HC-Pro* plant lines addressed the possibility of transcriptional gene silencing (TGS) of the three transgenes involved, all of which are under the control of the CaMV 35S promoter. This was especially important because it has been shown that T-DNA insertion mutants that carry 35S promoter sequences, such as the *rav2* knockout line used in this work, can induce TGS of other 35S promoters in the genome [36] and because HC-Pro cannot suppress silencing at the transcriptional level [37],[38]. RNA gel blot analysis showed that the level of HC-Pro mRNA was similar in all plants carrying the HC-Pro transgene (Fig. 4B), arguing against transcriptional silencing of 35S promoter sequences in the plants. In addition, the presence of siRNAs that derive from the GUS transcripts (Fig. 4C) indicates that the observed silencing of the GUS transgenes is at the post-transcriptional rather than the transcriptional level.

The role of RAV2 in HC-Pro suppression of hairpin transgene silencing was assayed using northern blot analysis to measure the accumulation of *6b4* GUS target mRNA as well as that of GUS primary and secondary siRNAs (Fig. 4C). As previously reported [7], HC-Pro blocked target RNA degradation when *306/6b4/HC-Pro* transgenic plants were wild type for *RAV2*, showing the characteristic absence of secondary siRNAs accompanied by high levels of nonfunctional primary siRNAs (Fig. 4C, compare lanes 3 and 4). In contrast, HC-Pro failed to prevent degradation of the *6b4* GUS mRNA target in the *rav2* knockout background (Fig. 4C, lanes 1 and 2). In addition, accumulation of GUS primary siRNAs was reduced in the *rav2* compared to the *RAV2* background and was similar to that in *306/6b4* plants without HC-Pro (Fig. 4C, lanes 1–4). Accumulation of secondary siRNAs, which are diagnostic of transitive silencing, was suppressed in HC-Pro transgenic plants even in the *rav2* knockout background (Fig. 4C, lanes 1–3), suggesting that HC-Pro-suppression of transitive silencing is RAV2-independent. In this experiment, however, we cannot rule out the possibility that the *rav2* knockout itself eliminates accumulation of secondary siRNAs. Therefore, our results suggest that RAV2 is required for the HC-Pro-mediated block in primary siRNA activity, but not for HC-Pro suppression of transitive silencing.

### *RAV2* is required for suppression of hairpin transgene-induced silencing by the carmovirus suppressor of silencing, P38

To determine if *RAV2* plays a general role in viral suppression of silencing, we used the *306*/*6b4* hairpin transgene silencing system to investigate whether Turnip Crinkle Virus (TCV) P38, a viral suppressor of silencing from a different virus family than TuMV HC-Pro [39], requires RAV2 to block silencing. The *rav2* knockout line was crossed to a *306*/*6b4* line that expresses P38, and the resultant F_1_ plants were allowed to self-fertilize. F_2_ plants were genotyped, and individuals containing the *306/6b4/P38* loci in the homozygous *rav2* mutant background were identified along with control plants containing all three loci in the *RAV2* background.

We used northern blot analysis to confirm the expected pattern of expression of *RAV2* and *P38* in these two sets of plants (Fig. 4D) and to examine suppression of silencing by P38 in the presence and absence of RAV2. Previous experiments showed that P38 behaves much like HC-Pro in the *306/6b4* transgene silencing system, blocking silencing and allowing *6b4* GUS mRNA to accumulate, even though high levels of GUS primary siRNAs also accumulate [7]. Similar to HC-Pro, P38 also blocks transitive silencing in this system as indicated by the absence of GUS secondary siRNAs [7]. In the current work, *P38* transgenic *306/6b4* plants with at least one copy of the wild type *RAV2* locus replicated those earlier results, showing P38 suppression of silencing, with a concomitant increase in accumulation of GUS primary siRNAs and elimination of GUS secondary siRNAs (Fig. 4E, compare lanes 3 and 4). In contrast, P38 suppression of silencing was strongly diminished in the *rav2* knockout background (Fig. 4E, lanes 1 and 2). Similar to our results with HC-Pro, accumulation of primary siRNAs in plants expressing P38 was much reduced in the *rav2* compared to the *RAV2* background, whereas secondary siRNA accumulation was unaffected by the loss of RAV2 and remained undetectable (Fig. 4E, compare lanes 1 and 2 with lane 3). The variability in accumulation of primary siRNAs observed in the *rav2* background (Fig. 4E, lanes 1 and 2) probably reflects the facts that individual plants were tested and accumulation of primary siRNAs is greatly reduced, but not eliminated in the absence of RAV2. Altogether our results indicate that *RAV2* plays similar roles in suppression of silencing by P38 and HC-Pro. Interestingly, in both cases, RAV2 function is required for suppression of primary siRNA-directed target degradation, but dispensable for the block to transitive silencing.

### *RAV2* is required for some of the phenotypic defects induced by HC-Pro, but not for HC-Pro-mediated defects in the miRNA pathway

**Figure 5:**
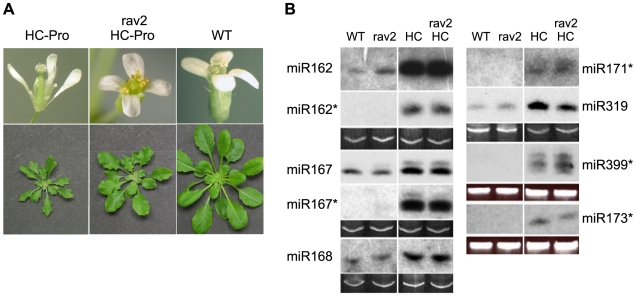
RAV2 is Required for Many HC-Pro-associated Morphological Anomalies but not for Defects in MicroRNA Biogenesis. (**A**) Flower morphological defects in HC-Pro transgenic plants (top left panel) are rescued in the *rav2* knockout background (top middle panel) resulting in flower phenotype indistinguishable from wild type (top right panel). Rosette dwarfing and leaf serration in transgenic plants (bottom left panel) are partially rescued in the rav2 knockout background (bottom middle panel) resulting in a phenotype intermediate between wild type (bottom right panel) and Hc-Pro plants. (**B**) The accumulation of the indicated miRNAs and miRNA*s was determined from RNA gel blot analysis of low molecular weight RNA from wild type (WT), *rav2* knockout plants (rav2), HC-Pro plants (HC) and HC-Pro plants in the *rav2* knockout background (rav2, HC). Ethidium bromide (EtBr) staining of the predominant RNA species in the low molecular weight fraction is shown as a loading control.

*Arabidopsis* plants expressing TuMV HC-Pro display a number of developmental anomalies: the plants are dwarfed with serrated leaves and have abnormal flower morphology associated with severely reduced fertility (Fig. 5A; [14],[33]). The phenotype of homozygous *rav2* knockout plants, however, is indistinguishable from that of wild type plants (data not shown). To determine if *RAV2* is required for any of the HC-Pro associated developmental anomalies, we compared the phenotype of HC-Pro plants in the wild type *RAV2* background to that of plants expressing approximately equal levels of HC-Pro mRNA, but in the *rav2* knockout background. The HC-Pro-mediated defects in flower morphology and fertility are completely alleviated in the absence of RAV2 (Fig. 5 and data not shown). In addition, both the dwarfing and serrated leaf phenotypes are mitigated - but not eliminated - in the *rav2* knockout background, resulting in an intermediate phenotype that is most visible when the plants are young (Fig. 5A), but becomes less distinguishable from that of wild type after the plants have flowered (Fig. 3A, 5A, and data not shown). These observations indicate that *RAV2* is required for HC-Pro-mediated flower and fertility defects and contributes to the defects in plant size and leaf shape.

In addition to its role in suppression of silencing, HC-Pro also causes defects in the biogenesis and function of certain endogenous small RNAs, including miRNAs, a class of small regulatory RNAs that plays critical roles in development. MiRNAs arise by processing of stem-loop primary transcripts by a Dicer-like enzyme, usually DCL1. The initial product is a 21-nt duplex, composed of the mature miRNA and the imperfectly complementary opposite strand, which is called miRNA*. The two strands separate and the mature miRNA binds to an AGO protein, forming the core of the miRNA effector complex. In HC-Pro transgenic plants, the level of many miRNAs is increased, often dramatically [33],[40]. Despite the increased level of the miRNA in the HC-Pro plants, the miRNA-targeted messenger RNAs also show an increased accumulation, suggesting that the miRNAs have reduced function [33],[41]. In addition, the miRNA* strand, which is unstable and fails to accumulate in wild type plants, characteristically accumulates to high levels in HC-Pro transgenic plants [33]. Together these results have led to the idea that HC-Pro impedes the proper separation of the strands of the miRNA:miRNA* duplex, leading to reduced association of the mature miRNA with AGO and thereby reducing miRNA function.

Because *RAV2* is required for HC-Pro effects on the biogenesis and function of primary siRNAs, as well as for many of the HC-Pro-associated developmental anomalies, we hypothesized that *RAV2* might also be required for HC-Pro-mediated defects in the miRNA pathway. To address the role of *RAV2* in HC-Pro-associated defects in miRNA biogenesis, we compared the levels of a variety of miRNAs and their corresponding miRNA* strands in HC-Pro plants in the presence and absence of RAV2. In all cases, the levels of miRNA and miRNA* were independent of RAV2 (Fig. 5B). These results indicate that *RAV2* is not required for the HC-Pro-associated defects in miRNA biogenesis.

To determine if RAV2 is involved in HC-Pro-associated defects in miRNA function, we compared the levels of a set of known miRNA-targeted messenger RNAs in *RAV2*/HC-Pro plants to those in *rav2*/HC-Pro plants using whole genome tiling microarray data (see following section for details of the tiling array experiments). Because HC-Pro interferes with the activity of some miRNAs [33],[41], we expected the tiling array data to show increased expression of at least some miRNA-targeted genes in HC-Pro plants. The tiling array data supported this expectation. Specifically, out of 146 verified miRNA targets [42],[43],[44],[45], we found that 39 showed altered expression in the HC-Pro transgenic line compared to the wild type control. Of these, 35 had increased expression, and only one of these was up-regulated in HC-Pro/*RAV2* versus HC-Pro/*rav2* plants (Table S1), suggesting that RAV2 does not play a general role in HC-Pro inhibition of miRNA activity. Altogether, the results suggest that, although RAV2 is required for many of the morphological anomalies in HC-Pro transgenic plants, it is not required for the HC-Pro-mediated defects in either the biogenesis or function of miRNAs.

### Whole genome tiling analysis links HC-Pro suppression of silencing to the network of host defense pathways

**Figure 6:**
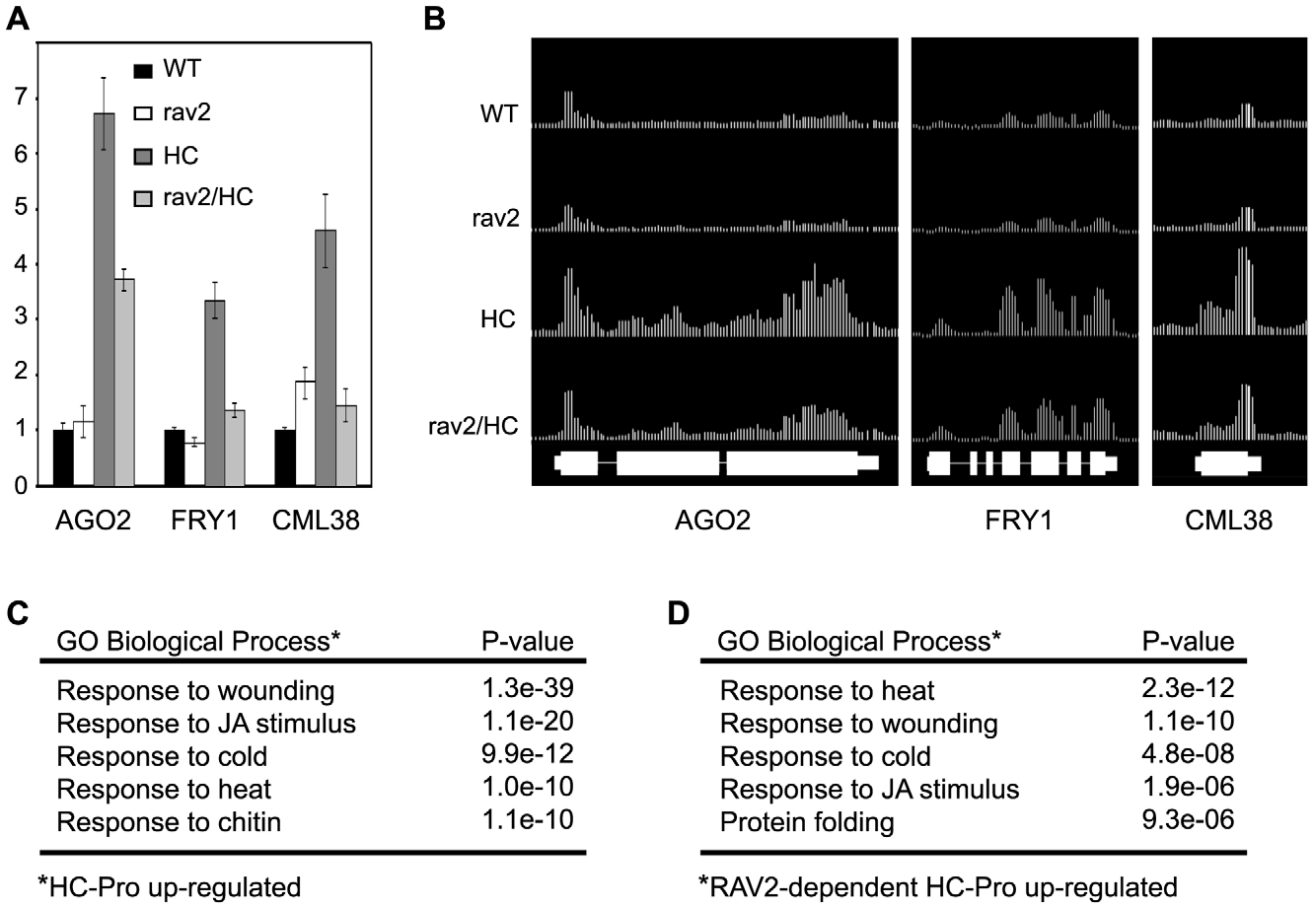
Tiling Microarray Analysis and RT qPCR Show *RAV2*-dependent Up-regulation of Silencing-associated Genes by HC-Pro. (**A**) The mRNA levels for *AGO2* (At1g31280), *FRY1* (At5g63980) and *CML38* (At1g76650) in *rav2* knockout plants (rav2), HC-Pro transgenic plants (HC), HC plants in the *rav2* knockout background (rav2/HC) and wild type control plants (WT) were determined by oligo(dT)-primed RT qPCR analysis. Error bars, ±SD. (**B**) The mRNA levels for the same genes shown in (A) were determined by *Arabidopsis* whole-genome tiling microarray expression analysis. The top four tracks show the level of these mRNAs in the genotypes indicated to the left of the track. The bottom track indicates the annotated gene models for the three loci. (**C**) Gene ontology (GO) analysis results for genes that are up-regulated in HC-Pro transgenic plants as compared to wild type plants. The top five over-represented biological processes categories and the associated hypergeometric distribution P-values are shown. (**D**) GO analysis results for genes that are up-regulated by HC-Pro in a RAV2-dependent manner. The top five over-represented biological processes categories and the associated hypergeometric distribution P-values are shown.

Because RAV2 is a transcription factor, we expected that it might be required for some HC-Pro-mediated changes in gene expression and that identifying these genes could provide insight into the role of RAV2 in HC-Pro suppression of silencing. To address this idea, we employed whole genome tiling microarray experiments to determine if the global pattern of gene expression is altered in HC-Pro transgenic plants and, if so, whether any of the changes are dependent on RAV2 function. *Arabidopsis* plants with four different genotypes were used in this experiment: 1) a *rav2* mutant line, 2) an HC-Pro expressing line, 3) the *rav2* mutant line expressing HC-Pro, and 4) the wild type (Columbia ecotype) control. We grew all four genotypes under identical conditions, extracted total RNA from plants just before bolting and used poly-A RNA to generate probes for hybridization to the *Arabidopsis* tiling arrays as previously described [46],[47]. TileMap [48] was used to identify genes that are significantly up- or down-regulated in each line as compared to wild type plants, as well as to compare the pattern of gene expression in *RAV2*/HC-Pro plants versus *rav2*/HC-Pro plants (Tables S2–S9). To check the tiling results, the expression of ten genes in these plant lines was additionally examined using real-time quantitative PCR (RT qPCR). This analysis confirmed the relative levels of expression of these genes determined by the tiling array in 33 of 40 two-way comparisons between the four genotypes (Fig. 6A and B and Fig. S2).

One of the first questions we addressed was whether genes involved in antiviral silencing and other small RNA pathways were affected by HC-Pro and RAV2. Unexpectedly, none of the genes encoding components of the silencing machinery or otherwise known to be required for silencing were down-regulated in the HC-Pro plants. Expression of *RAV2* itself was also not altered in HC-Pro plants. However, a number of silencing-associated genes were up-regulated in HC-Pro plants. The up-regulated genes included three of the ten *Arabidopsis AGO* family members, *AGO2*, *AGO3*, and *AGO4*. *AGO4* is required for some kinds of transcriptional silencing. The roles of *AGO2* and *AGO3* are unknown, but neither has been associated with antiviral silencing [49],[50]. Interestingly, two genes implicated as endogenous suppressors of silencing were also up-regulated in HC-Pro: Arabidopsis *FIERY1* (*FRY1*), which negatively regulates transitive silencing [51], and *CML38* (At1g76650), which is a likely *Arabidopsis* homolog of *rgsCaM*, an endogenous suppressor of antiviral silencing in tobacco [23]. Like *RAV2*, *rgsCaM* was originally identified as an HC-Pro interacting protein [23]; however, it is not yet known whether *rgsCaM* is required for HC-Pro to suppress silencing. RT qPCR confirmed the relative expression levels of *AGO2*, *FRY1*, and *CML38* in the HC-Pro expressing line compared to wild type plants (compare Fig. 6A and 6B). The RT qPCR data also showed that increases in both *FRY1* and *CML38* expression required *RAV2*, whereas the increase in *AGO2* expression was only partially dependent on *RAV2* (Fig. 6A). These results argue that the mechanism for HC-Pro suppression of silencing does not involve down-regulation of genes required for silencing, but rather a RAV2-dependent up-regulation of genes that potentially antagonize antiviral silencing.

The tiling array analysis was used to identify global HC-Pro-mediated changes in gene expression and determine which, if any, depended on RAV2. A significant number of genes were differentially regulated in the HC-Pro plants; 2580 were up-regulated (Table S2) and 2060 were down-regulated (Table S3). Many fewer genes were differentially affected in *RAV2*/HC-Pro compared to *rav2*/HC-Pro plants (Tables S4 and S5). Of 265 genes that showed dependence on RAV2 for up-regulation by HC-Pro (Table S10), only a small number showed changed expression in *rav2* mutant plants in the absence of HC-Pro as compared to wild type (20 of 265 were up-regulated; 17 of 265 were down-regulated). Similarly, of 433 genes that showed dependence on RAV2 for down-regulation by HC-Pro (Table S11), a relatively small number showed changed expression in the *rav2* knockout plants in the absence of HC-Pro as compared to wild type (15 of 433 were up-regulated; 98 of 433 were down-regulated). Together, these results suggest that HC-Pro causes major changes in global gene expression patterns, some of which are mediated by RAV2. Interestingly, based on comparison of the set of genes with altered expression in *rav2* mutant plants with the set altered by HC-Pro in a *RAV2*-dependent manner, it appears that HC-Pro changes the scope and spectrum of genes that are controlled by *RAV2*.

Gene Ontology (GO) term analysis was used to give a functional characterization of the tiling array results [52]. A key finding of this analysis was that multiple stress and defense responses were induced in HC-Pro expressing plants. The top four biological process categories that were over-represented among genes up-regulated in HC-Pro compared to wild type plants were: response to wounding (67 of 119 genes), response to jasmonic acid (JA) stimulus (48 of 119 genes), cold stress (49 of 197 genes) and heat stress (33 of 109 genes) (Fig. 6C). Strikingly, genes in these same four categories were also over-represented among the genes that are up-regulated by HC-Pro in a RAV2-dependent manner (Fig. 6D). Tables showing the specific genes that are up-regulated by HC-Pro in each of these GO categories, as well as the subsets that require RAV2 for HC-Pro up-regulation are in the Supplementary Tables (Tables S12–15). These results indicate that RAV2 plays a role in altered expression of stress and defense pathways in HC-Pro plants. Interestingly, *FRY1* and *CML38*, both of which have been implicated as suppressors of silencing [23],[51] and are induced by HC-Pro in a RAV2-dependent manner (Fig. 6B), have GO annotations of response to cold and wounding, respectively, suggesting a link between silencing and other stress and defense pathways.

## Discussion

It has been over a decade since the first plant viral suppressors of RNA silencing were reported [53],[54],[55], providing an early clue that silencing serves as an anti-viral defense in plants and leading to the identification of many other such silencing suppressors [56]. However, the mechanisms by which these viral proteins manipulate silencing have remained largely elusive. Here we report the identification of a host protein, the transcription factor RAV2, that is required for suppression of silencing mediated by two unrelated viral proteins, potyviral HC-Pro and carmoviral P38. *RAV2* is part of a gene family that comprises six members, two of which (*RAV1*; At1g13260 and *RAV2-like*; At1g25560) are very closely related to *RAV2*. Surprisingly, however, neither of these related genes is able to compensate for the loss of *RAV2* with respect to suppression of silencing mediated by either HC-Pro or P38. This result indicates that RAV2 provides a unique function in suppression of silencing. The identification of RAV2 as an important element in viral suppression of silencing provides a handle for identifying additional host partners and thereby unraveling the pathway of host involvement in that process.

The discovery that plant viruses from many unrelated families encode suppressors of silencing has underscored the importance of silencing in antiviral defense. Similarly, we expect our finding that viral suppressors from two unrelated viruses have evolved independently to require RAV2 underscores the importance of host proteins in viral counter-defense. In addition, it suggests that RAV2 represents an effective and readily subverted control point – either for suppression of silencing in general or for a subset of suppressors with some mechanistic features in common. It will be interesting to see how general the requirement for RAV2 is in viral suppression of silencing.

How could a transcription factor such as RAV2 be used to suppress silencing? Two reports have identified RAV2 as a repressor of at least some target genes [57],[58]. Therefore, it seemed reasonable to hypothesize that the role of RAV2 in HC-Pro suppression of silencing is to repress transcription of genes that encode components of the silencing machinery for the anti-viral branch of the silencing pathway. However, our global analysis of genome expression indicates that the expression of genes known to be required for RNA silencing is unchanged in HC-Pro transgenic plants as compared to wild type controls. Instead, our data shows that RAV2 is required for HC-Pro-mediated up-regulation of some stress and defense response genes. Earlier work showing that induction of both biotic and abiotic stresses interferes with RNA silencing induced by a viral amplicon in tobacco is consistent with a mechanism in which induction of other defense responses can divert the host from antiviral silencing [59]. The observation that *RAV2* is induced by the ethylene defense pathway and is also required for viral suppression of silencing emphasizes the importance of crosstalk among defense pathways and supports the idea that RAV2 constitutes an important control point for the integration of defense responses during virus infection.

One puzzle raised by the observation that HC-Pro, which is a cytoplasmic protein [60],[61], interacts with a host transcription factor is: How and where do the two proteins have the opportunity to meet? Although HC-Pro has been shown to accumulate in nuclear inclusions in certain potyviral infections, it is thought that such inclusions represent storage of excess protein [61]. Thus, it seems more likely that HC-Pro and RAV2 interact in the cytoplasm. Sequestering transcription factors in the cytoplasm is a common mechanism used in eukaryotic organisms for controlling the activity of such proteins [62],[63]. The interaction of HC-Pro with RAV2 in the cytoplasm could either reflect a direct involvement of RAV2 itself in suppression of silencing or interference by HC-Pro in the cellular control of RAV2 – either to block activation or promote inappropriate activation – thereby changing host gene expression in such a way that promotes suppression of silencing. Elucidating these issues, as well as examining whether P38 also physically interacts with RAV2, is likely to be a fruitful area of research.

Another particularly interesting aspect of our results is the differential requirement for RAV2 in suppression of different small RNA-mediated processes. Both HC-Pro and P38 suppress transitive silencing in the absence of RAV2; yet, both suppressors require RAV2 for suppression of target degradation via the activity of primary siRNAs. Furthermore, although HC-Pro requires RAV2 to block the activity of primary siRNAs, RAV2 is not required for HC-Pro-mediated defects in miRNA activity. Our present work does not distinguish whether these differential requirements for RAV2 indicate a fundamental difference in the mechanisms responsible for suppression of these processes or simply a difference in the cofactor requirements of a common mechanism.

One current model for viral suppression of small RNA pathways posits a general mechanism in which small RNA duplexes are bound by the suppressor, thereby blocking the incorporation of one strand of the duplex into an active effector complex [17],[64]. Our data showing a role for RAV2 in suppression of silencing does not directly support this proposed mechanism, but is also not inconsistent with it. Indeed, it has been shown that small RNA binding by HC-Pro in vitro is enhanced by unknown cellular factors [17],[64]. Thus, RAV2 might be one such factor, acting either directly or indirectly to enhance small RNA binding.

Expression of HC-Pro in transgenic plants causes a set of morphological anomalies that have been attributed to defects in the biogenesis and function of endogenous miRNAs [33]. However, there is emerging evidence that suggests that the phenotypic changes are largely independent of the miRNA pathway [14],[15],[20],[65]. In support of this notion, the data we have presented here indicate that many of the HC-Pro-mediated morphological anomalies are RAV2-dependent whereas the defects in the miRNA pathway are RAV2-independent, arguing against a causative role for miRNAs in most HC-Pro-associated morphological anomalies

Although the mechanism by which HC-Pro uses RAV2 to suppress silencing is not yet clear, the results of our tiling array analysis suggest two interesting, though speculative, possibilities. The first of these relates to the induction of *AGO2* and a subset of other *AGO* genes in HC-Pro transgenic plants, an effect that is only partially dependent on *RAV2*. The *AGO* genes that are up-regulated by HC-Pro are not required for post-transcriptional gene silencing (PTGS). These results suggest that an alteration of the mix of AGO proteins in the cell might tip the balance away from PTGS towards other small RNA pathways that are not directly involved in anti-viral defense. The recent demonstration that changing the 5′ nucleotide of a miRNA so as to favor binding to AGO2 instead of AGO1 inactivates that miRNA [66] supports the idea that an overabundance of the wrong AGO proteins could contribute to suppression of silencing. The second interesting possibility suggested by our tiling data concerns the result that HC-Pro requires RAV2 to induce expression of *FRY1* and *CML38*, both of which have been implicated as endogenous suppressors of silencing and both of which are associated with stress or defense responses. Induction of endogenous suppressors of silencing may be more widespread than we know because most have probably not yet been identified [51]. It is tempting to speculate that the induction of stress and defense pathways by HC-Pro might have the counter-productive result - from the plant's perspective - of inducing a set of endogenous suppressors of antiviral silencing.

## Materials and Methods

### Plant Material and Transgenic Lines

The tobacco *6b5* [32] and Arabidopsis *TuMV HC-Pro [CT25* [14] *]*, *TCV-P38* [39], *306* and *6b4* [35] lines have been previously described. The Arabidopsis *rav2/edf2* (At1g68840) T-DNA insertion line (SALK_070847) was used and did not express detectable levels of RAV2 mRNA as assayed by northern analysis. See Text S1 for the procedures used to generate the *35S:ntRAV* tobacco transgenic line and genotyping of the SALK_070847 T-DNA insertion line. All Arabidopsis plants were of the Columbia ecotype.

### GUS Histochemical Staining

Histochemical staining for GUS activity was carried out as described [53].

### VIGS Silencing Assays

The silencing of endogenous *CH42* expression using the geminivirus CaLCV vector was performed exactly as described previously [14].

### RNA Isolation and Northern Analysis

RNA isolation and RNA gel blot analysis of high and low molecular weight RNA were performed exactly as previously described [14],[40],[67]. Probes for detection of TuMV HC-Pro, TCV-P38 and 6b4 mRNAs, miRNA as well as those for primary and secondary siRNAs from the *6b4/306* transgene silencing system were previously described [7]. The *RAV2* probe was generated using the primer set (5′ primer-TTGGAAAGTTCGGTCTGGTC and 3′ primer-TAATACGACTCACTATAGGGACCGCAAACATATCATCAACATCTC), which generate a 152 bp fragment from the 3′ end of the gene. The 3′ RAV2 primer contains T7 promoter sequences and a 4 nucleotide spacer at its 5′ end to facilitate synthesis of the probe using T7 polymerase.

### GST Pulldown Assays

The production of the HC-Pro-GST fusion protein and ^35^S-methionine labeled ntRAV is described in Text S1. To determine if HC-Pro-GST and ntRAV interact, approximately equimolar amounts of GST or HC-Pro-GST fusion protein were added to 20 µl of glutathione sepharose 4B beads (GE Healthcare) in GLB buffer (50 mM Tris-HCl, pH 8.0, 150 mM NaCl, 1 mM EDTA, and 1 mM PMSF) supplemented to contain 100 µg/ml BSA and 0.1% NP-40 (Roche) and shaken gently for 1 hour at 4°C. After rinsing with supplemented GLB, an equal amount of ^35^S-methionine labeled ntRAV was added to each sample, shaken gently at 4°C for 2 hours and rinsed again with supplemented GLB. Bound protein was eluted from the beads with Laemmli sample buffer, resolved by SDS-PAGE, and transferred to PVDF membrane. ^35^S-methionine labeled ntRAV was visualized by autoradiography.

### Co-immunoprecipitation of RAV2 and HC-Pro

Protein was extracted from 0.5 g of Arabidopsis rosette leaf tissue by the following procedure. Tissue was frozen in liquid nitrogen, ground into powder with a mortar and pestle, homogenized in 4 ml of protein extraction buffer (40 mM Tris-Cl, pH 8.0, 200 mM NaCl, 2.5 mM EDTA, 1% Triton X-100, 0.1% NP-40) containing protease inhibitor cocktail (Roche), and centrifuged (12,000 g at 4°C). The supernatant was incubated with 100 µl pre-washed anti-FLAG M2 agarose beads (Sigma F2426) at 4°C for two hours. Agarose beads containing protein complexes were washed three times with extraction buffer, boiled in SDS sample buffer, resolved on a 10% SDS polyacrylamide gel, and subjected to western blotting. The presence of RAV2 protein was detected using a rabbit anti-RAV2 peptide antibody generated from the peptide GGKRSRDVDDMFALRC, and a rabbit anti-HC-Pro peptide antibody generated from the peptide KEFTKVVRDKLVGE was used to detect HC-Pro. Both RAV2 and HC-Pro peptide antibodies were produced by Sigma-Genosys.

### Tiling Microarray Analysis

Total RNA was isolated as described above from the above ground portions of six week old plants that had not yet bolted. Generation of probes to poly-A RNA and hybridization to the tiling arrays were performed as described previously [46],[47]. The data was analyzed using the program TileMap with a posterior probability of 0.8 [48]. The TileMap program identifies sequences that have significant changes in expression compared to controls, but does not provide fold-differences in expression levels. GO analysis was performed using ProfCom [68].

## Supporting Information

Text S1 Supplementary experimental procedures
(0.07 MB PDF)

Figure S1 Protein gel for estimating GST and HC-Pro-GST relative concentrations. Samples containing the indicated volumes (vol) of GST and HC-Pro-GST were resolved by SDS-PAGE, and the proteins were visualized by staining with Coomassie blue. Kaleidoscope Precision Plus prestained protein standards (Biorad) were used as the size markers (lane 4), and the sizes are indicated above each band.
(0.35 MB PDF)

Figure S2 Comparison of Tiling Microarray and RT qPCR Analyses. (A) The mRNA levels for the seven indicated genes in *rav2* knockout plants (rav2), HCPro transgenic plants (HC), HC plants in the *rav2* knockout background (rav2/HC) and wild type control plants (WT) were determined by oligo(dT)-primed RT qPCR analysis. Error bars, ±SD. (B) The mRNA levels for the same genes shown in (A) were determined by *Arabidopsis* whole-genome tiling microarray expression analysis. The top four tracks show the level of these mRNAs in the genotypes indicated to the left of the track. The bottom track indicates the annotated gene models for the three loci.
(0.11 MB PDF)

Table S1 Tiling array results: miRNA target genes whose expression is altered in HC-Pro plants
(0.02 MB XLS)

Table S2 Tiling array results: Arabidopsis genomic regions up-regulated in HC-Pro compared to WT plants
(0.72 MB XLS)

Table S3 Tiling array results: Arabidopsis genomic regions down-regulated in HC-Pro compared to WT plants
(0.57 MB XLS)

Table S4 Tiling array results: Arabidopsis genomic regions up-regulated in HC-Pro compared to rav2/HC-Pro plants
(0.09 MB XLS)

Table S5 Tiling array results: Arabidopsis genomic regions down-regulated in HC-Pro compared to rav2/HC-Pro plants
(0.16 MB XLS)

Table S6 Tiling array results: Arabidopsis genomic regions up-regulated in rav2/HC-Pro compared to WT plants
(0.48 MB XLS)

Table S7 Tiling array results: Arabidopsis genomic regions down-regulated in rav2/HC-Pro compared to WT plants
(0.24 MB XLS)

Table S8 Tiling array results: Arabidopsis genomic regions up-regulated in rav2 compared to WT plants
(0.11 MB XLS)

Table S9 Tiling array results: Arabidopsis genomic regions down-regulated in rav2 compared to WT plants
(0.19 MB XLS)

Table S10 Tiling array results: Arabidopsis genomic regions up-regulated in HC-Pro compared to rav2/HC-Pro and WT plants
(0.05 MB XLS)

Table S11 Tiling array results: Arabidopsis genomic regions down-regulated in HC-Pro compared to rav2/HC-Pro and WT plants
(0.07 MB XLS)

Table S12 Tiling array results for the GO category, response to wounding: genes that are up-regulated in HC-Pro compared to WT plants. Yellow indicates the subset up-regulated in HC-Pro compared to rav2/HC-Pro plants.
(0.02 MB XLS)

Table S13 Tiling array results for the GO category, response to JA stimulus: genes that are up-regulated in HC-Pro compared to WT plants. Yellow indicates the subset up-regulated in HC-Pro compared to rav2/HC-Pro plants.
(0.02 MB XLS)

Table S14 Tiling array results for the GO category, response to cold: genes that are up-regulated in HC-Pro compared to WT plants. Yellow indicates the subset up-regulated in HC-Pro compared to rav2/HC-Pro plants.
(0.02 MB XLS)

Table S15 Tiling array results for the GO category, response to heat: genes that are up-regulated in HC-Pro compared to WT plants. Yellow indicates the subset up-regulated in HC-Pro compared to rav2/HC-Pro plants.
(0.02 MB XLS)
