# Supplementary material for: Two Plant Viral Suppressors of Silencing Require the Ethylene-Inducible Host Transcription Factor RAV2 to Block RNA Silencing
Source: PLoS Pathog. 2010 Jan 15;6(1):e1000729. doi: 10.1371/journal.ppat.1000729 (PMC2800190; doi:10.1371/journal.ppat.1000729)
Supplement: Text S1 — Supplementary experimental procedures (0.07 MB PDF) [file ppat.1000729.s001.pdf]

SUPPLEMENTARY EXPERIMENTAL PROCEDURES

Genotyping the *rav2/edf2* T-DNA insertion line and HC-Pro expressing plants

The *rav2/edf2* (At1g68840) T-DNA insertion line (SALK_070847) was genotyped using the following primers: 5' primer, GTGTTGTTCCTCAGCCTAACG; 3' primer, TTTCCACAAAACCATTGTTCC; and the T-DNA specific primer GCGTGGACCGCT TGCTGCAACT. The 5' primer and 3' primer set flanked the insertion site and amplified a 1000 bp fragment from the wild type locus, and the 3' primer and the T-DNA specific primer set amplified a 500 bp fragment from the SALK_070847 chromosome. To identify lines containing the HC-Pro transgene we used a primer set (5' primer, GTGCCCAGAAGTTCAAGAGC and 3' primer, ACGACTATGCCACTCCAACC) that amplifies a 510 bp fragment. HC-Pro plants were also identified based on their characteristic phenotype [1,2].

Generation of the *35S:ntRAV* tobacco transgenic line

ntRAV cDNA was amplified using the following primer set:

5' primer, GCTTAATTAAGGCGCGCCGAAAATGGAAGGTAGCAGC and 3' primer, GCGGATCCATTTAAATGTTACTATTACATGTTACAAGGC. The amplified cDNA was cloned into the Asc1 and BamH1 sites of the plant binary vector *p*FGC-1008 plasmid (AY310333) [3], and transferred to *Agrobacterium* strain GV3101. The construct was then transformed into *Nicotiana tabacum* (cv *xanthi*) using *Agrobacterium*-mediated transformation.

Generation of a *FLAG-RAV2*-expressing Arabidopsis line

To generate an *Arabidopsis thaliana* line expressing an N-terminal FLAG-epitope tagged RAV2 (RAV2-FLAG), *RAV2* cDNA was amplified using the primer set:

5’ primer, CACCATGGATTCTAGTTGCATAGACGAG and 3’ primer, TCACAAAGCATTGATTATCGCCTGCTTCTTG. The amplified cDNA was cloned into the Invitrogen pENTR™ entry vector following the manufacturer’s instructions and subsequently into the pEarleyGate 202 binary vector using the Invitrogen LR Closase II plus enzyme mix [4]. The plasmid was transferred into *Agrobacterium* strain GV3101, and plants containing a T-DNA insertion in the *RAV2* gene (SALK_070847) were transformed using the *Agrobacterium* floral dip method. Transformants were selected based on their BASTA resistance. Plants expressing both RAV2-FLAG and HC-Pro were generated by crossing plants carrying the *RAV2-FLAG* transgene to those carrying the TuMV *HC-Pro* transgene.

GST-fusion protein production, purification, and quantitation

To generate an HC-Pro-GST epitope tagged fusion protein, full length TEV HC-Pro was amplified using the primer set (5’ primer-CCCCAGCGACAAATCAATCTCTGA and 3’ primer-CCTCCAACATTGTAAGTTTTCA), ligated into the SmaI site of the pGEX-2TK expression vector (GE Healthcare), and transformed into *Escherichia coli* strain BL21(DE3*) cells. A 50 ml culture of cells carrying the HC-Pro-GST construct was grown in Luria-Burtani medium with 100 mg/ml ampicillin at 30°C and diluted into 2 L of the same medium and grown at 30°C for 3 hours. Expression of HC-Pro-GST was induced by addition of 1 mM isopropyl β-D-thiogalactopyranoside to a final concentration of 1 mM. After 3 hours at 30°C, cells were harvested by centrifugation, resuspended in GST lysis buffer (GLB: 50 mM Tris-HCl, pH 8.0, 150 mM NaCl, 1 mM EDTA, and 1 mM PMSF), and lysed by sonication. The cell lysate was incubated with glutathione sepharose 4B (GE Healthcare) overnight at 4°C. The GST fusion proteins were eluted for 1 hour at 4°C with 20 mM glutathione, 50 mM Tris-HCl, pH 8.0. GST was prepared in similar fashion from bacteria carrying the pGEX-2TK expression vector.

The relative concentrations of the GST and HC-Pro-GST preparations were estimated using Coomassie blue staining of the proteins after they were resolved by SDS-polyacrylamide gel electrophoresis (Fig. S1). A concentration series of the proteins was electrophoresed to insure that staining of at least some bands would be in the linear range and proportional to the amount of protein. Coomassie blue binds primarily to basic amino acids [5,6]. Therefore, based on the size of the proteins (GST, 239 amino acids; HC-Pro-GST, 458 + 239 amino acids), as well as on their actual amino acid compositions, we expect that an HC-Pro-GST band will stain about three times as intensely as a GST band containing an equimolar amount of protein. We estimated that staining of the sample containing 0.2 µl GST (Fig. S1 lane 1) was comparable to that of a sample containing 7 µl to 10 µl HC-Pro-GST (Fig. S1, compare lane 1 to lanes 5 and 6). Therefore, about 20 to 30 µl HC-Pro-GST and 0.2 µl GST should have comparable amounts of the GST moiety. We used 0.2 µl GST and 20 µl HC-Pro-GST in the pull-down assays.

To generate the ^{35}S-methionine labeled ntRAV protein that was used in the *in vitro* pull-down assays, the full-length *ntRAV* cDNA was amplified using the primer set (5' primer-GCGGCGCGCCCATAAAAAAAACAAAACA and 3' primer-GTGGCGCGCCTAAAAGAAAATGGAAGGT). The purified fragment was cloned into pCR4-TOPO using the TOPO TA Cloning® Kit (Invitrogen), and ^{35}S-methionine labeled protein was generated using the TNT™ Quick-coupled *in vitro* transcription/translation kit (Promega) according to manufacturer instructions.

RT qPCR

Total RNA for expression analysis by RT qPCR was isolated from the above ground portions of six-week old plants that had not yet bolted as described in Experimental Procedures. Experiments were performed using two biological replicates, each comprising RNA isolated

from five individuals of each genotype. RT qPCR was performed as previously described [7]. First strand cDNA was synthesized using 2 µg of total RNA and the Omniscript RT kit (Qiagen) according to manufacturer instructions. The cDNA reaction mixture was diluted 5-fold, and 5 µl was used as template in a 25 µl PCR reaction with iQ SYBR Green Supermix (BIO-RAD). Preincubation at 95º C for 3 min was followed by 40 cycles of denaturation at 95ºC for 30 sec, annealing at 55ºC for 30 sec, and extension at 72ºC for 30 sec. All the reactions were carried out on the BioRad iCycler iQ (BIO-RAD). The comparative threshold cycle (ct) method was used for determining relative transcript levels (Bulletin 5279, Real-Time PCR Applications Guide, BIO-RAD). *ACT2* was used as the internal reference, and expression levels were relative to the wild type. Primers were designed to amplify cDNA specifically using standard protocols as described [8] with regard to proximity to the 3' end, size of the amplified fragment and inclusion of an intron when possible to discriminate amplification of genomic DNA. The primer pairs used for each gene are listed below.

ACT2 (at3g18780)	5'-primer	GCTGTTGACTACGAGCAGGAGATGG
	3'-primer	GGCCTTGGAGATCCACATCTGCTGG
AGO2 (t1g31280)	5'-primer	GGTTTCTGAACCCGTTCGTGTAGCTG
	3'-primer	TACTCCACCTTGTATGACCCCGTAGG
AST12 (at3g51895)	5'-primer	GACAATACCAACTTTAACGCTCTCCTG
	3'-primer	GATTGGACTCGTCGTAGCGGTGGCG
CML38 (at1g76650)	5'-primer	GCATCATAAGAGCAAACTCATCAAAGC
	3'-primer	GAGGACAAGAACAGAGAGTTAGAGG

FRY1 (at5g63980)	5'-primer	ACTTGCCGTTAGCATCCATAGCAGG
	3'-primer	CCACCCGCCTCTGTAACAACTATAG
LOX3 (at1g17420)	5'-primer	TCCCTGCCGATCTAATTCGCAGAGG
	3'-primer	CTCCGGATGGGAGATGAAACTCGCG
SOT16 (at74100)	5'-primer	CCTCCACTAAGCCATCAATACGAGC
	3'-primer	GATGGTGTACATATGGAGAGACCCG
SS2 (at744020)	5'-primer	TGACTTCGCTGAGCAGAGTGTCTCC
	3'-primer	CTCACGCTTCTCCCCAATCCAAGTG
VTC (at4g26850)	5'-primer	CTGTTTCTCTGCGTAACACTGTGGC
	3'-primer	GTGTTGTTGCCATCAATGTTAGTCCG
WBC18 (at3g55110)	5'-primer	CGTCCTCTTATTCTTACTCCCGAAC
	3'-primer	CCGAATGTCATGATGAGTTACATGG
WRKY70 (at3g56400)	5'-primer	CATTGACGTAACTGGCCTGATGATG
	3'-primer	GTTTGAAGATTCCGCGATAGTCGG

REFERENCES

1. Kasschau KD, Xie Z, Allen E, Llave C, Chapman EJ, et al. (2003) P1/HC-Pro, a viral suppressor of RNA silencing, interferes with Arabidopsis development and miRNA function. Dev Cell 4: 205-217.
2. Mlotshwa S, Schauer SE, Smith TH, Mallory AC, Herr JM, Jr., et al. (2005) Ectopic DICER-LIKE1 expression in P1/HC-Pro Arabidopsis rescues phenotypic anomalies but not defects in microRNA and silencing pathways. Plant Cell 17: 2873-2885.
3. Kerschen A, Napoli CA, Jorgensen RA, Muller AE (2004) Effectiveness of RNA interference in transgenic plants. FEBS Lett 566: 223-228.
4. Earley KW, Haag JR, Pontes O, Opper K, Juehne T, et al. (2006) Gateway-compatible vectors for plant functional genomics and proteomics. Plant J 45: 616-629.
5. Compton SJ, Jones CG (1985) Mechanism of dye response and interference in the Bradford protein assay. Anal Biochem 151: 369-374.
6. Georgiou CD, Grintzalis K, Zervoudakis G, Papapostolou I (2008) Mechanism of Coomassie brilliant blue G-250 binding to proteins: a hydrophobic assay for nanogram quantities of proteins. Anal Bioanal Chem 391: 391-403.
7. Zhu J, Kapoor A, Sridhar VV, Agius F, Zhu JK (2007) The DNA glycosylase/lyase ROS1 functions in pruning DNA methylation patterns in Arabidopsis. Curr Biol 17: 54-59.
8. Udvardi MK, Czechowski T, Scheible WR (2008) Eleven golden rules of quantitative RT-PCR. Plant Cell 20: 1736-1737.
