# Supplementary figures and images for: Two Plant Viral Suppressors of Silencing Require the Ethylene-Inducible Host Transcription Factor RAV2 to Block RNA Silencing
Source: PLoS Pathog. 2010 Jan 15;6(1):e1000729. doi: 10.1371/journal.ppat.1000729 (PMC2800190; doi:10.1371/journal.ppat.1000729)

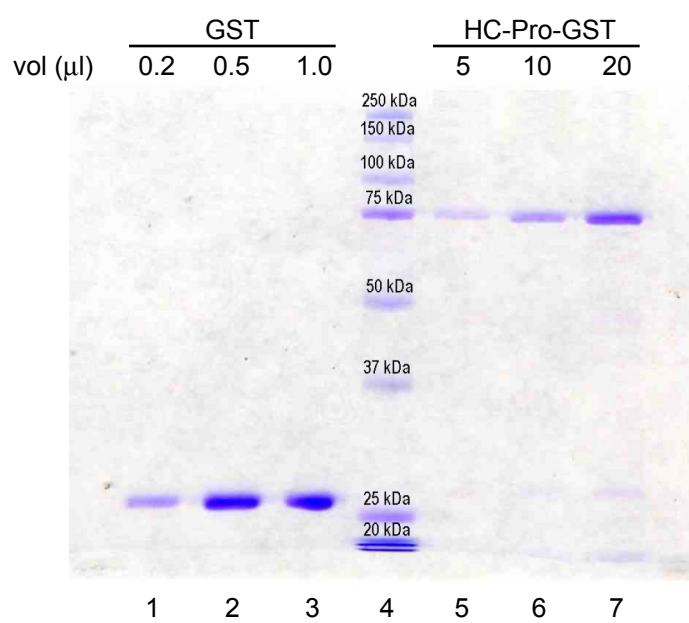

Supplement: Figure S1 — Protein gel for estimating GST and HC-Pro-GST relative concentrations. Samples containing the indicated volumes (vol) of GST and HC-Pro-GST were resolved by SDS-PAGE, and the proteins were visualized by staining with Coomassie blue. Kaleidoscope Precision Plus prestained protein standards (Biorad) were used as the size markers (lane 4), and the sizes are indicated above each band. (0.35 MB PDF) [file ppat.1000729.s002.pdf]

**A**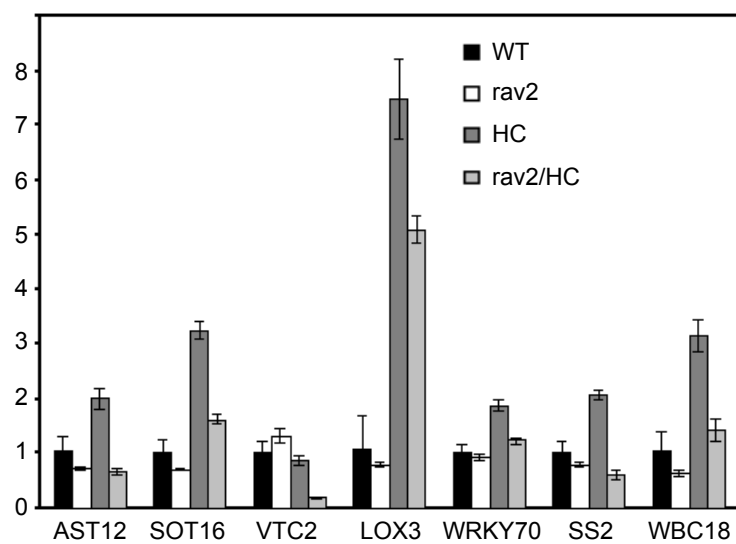**B**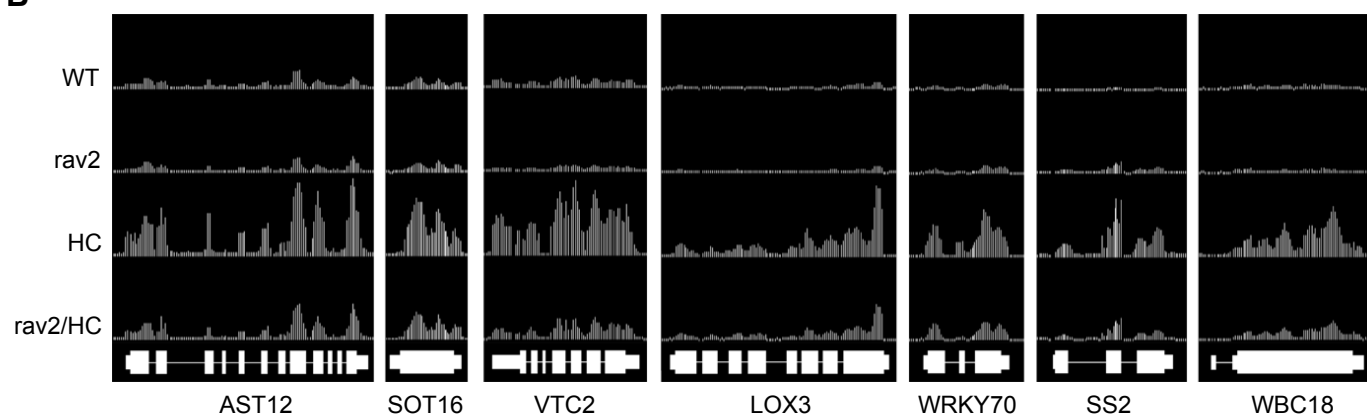

Supplement: Figure S2 — Comparison of Tiling Microarray and RT qPCR Analyses. (A) The mRNA levels for the seven indicated genes in rav2 knockout plants (rav2), HCPro transgenic plants (HC), HC plants in the rav2 knockout background (rav2/HC) and wild type control plants (WT) were determined by oligo(dT)-primed RT qPCR analysis. Error bars, ±SD. (B) The mRNA levels for the same genes shown in (A) were determined by Arabidopsis whole-genome tiling microarray expression analysis. The top four tracks show the level of these mRNAs in the genotypes indicated to the left of the track. The bottom track indicates the annotated gene models for the three loci. (0.11 MB PDF) [file ppat.1000729.s003.pdf]
